# Positional Fluorination of Fmoc-Phenylalanine Modulates Hydrogel Structure and Antibacterial Activity

**DOI:** 10.1021/acs.biomac.5c00481

**Published:** 2025-08-19

**Authors:** Ofir Doitch, Noam Rattner, Dana Cohen-Gerassi, Yoav Dan, Sigal Rencus-Lazar, Moran Aviv, Lihi Adler-Abramovich

**Affiliations:** † Department of Oral Biology, The Goldschleger School of Dental Medicine, Gray Faculty of Medical and Health Sciences, 26745 Tel Aviv University, Tel Aviv 6997801, Israel; ‡ Jan Koum Center for Nanoscience and Nanotechnology, Tel Aviv University, Tel Aviv 6997801, Israel; § The Center for the Physics and Chemistry of Living Systems, Tel Aviv University, Tel Aviv 6997801, Israel; ∥ Department of Materials Science and Engineering, Tel Aviv University, Tel Aviv 6997801, Israel; ⊥ School of Mechanical Engineering, 112886 Afeka Tel Aviv Academic College of Engineering, Tel Aviv 6910717, Israel

## Abstract

The rise of antibiotic-resistant bacteria emphasizes the urgent need for alternative therapeutic strategies. Self-assembling nanostructures, such as fluorenylmethoxycarbonyl-pentafluoro-l-phenylalanine (Fmoc-F_5_-Phe), have shown promising antibacterial activity by selectively targeting bacterial membranes. However, the influence of fluorine positioning on hydrogel’s physical and biological properties remains poorly understood. Here, we evaluate three single-fluorinated Fmoc-phenylalanine derivatives, each featuring a fluorine substitution at a different aromatic position. We demonstrate that even subtle positional changes dramatically affect self-assembly kinetics, nanostructure morphology, mechanical properties, and antibacterial performance. While Fmoc-F_5_-Phe hydrogels are not stable, the single-fluorinated analogues exhibit improved stability and mechanical properties. Among them, Fmoc-4-F-Phe shows the highest antibacterial activity, effectively inhibiting the growth of *Streptococcus mutans* at low concentrations, increasing ROS levels, disrupting bacterial morphology, and reducing biofilm formation. These biocompatible, self-assembled hydrogels offer a versatile platform for antimicrobial applications with potential in surface coatings, highlighting their promise as next-generation antibacterial biomaterials.

## Graphical abstract

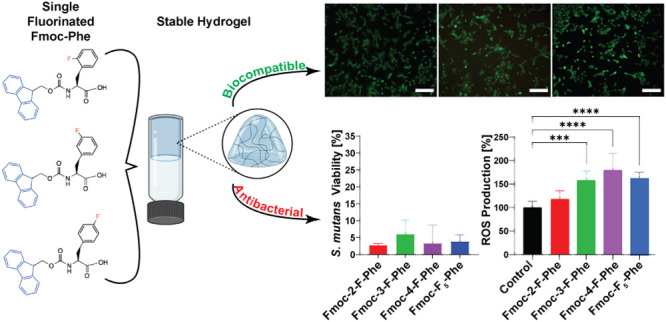

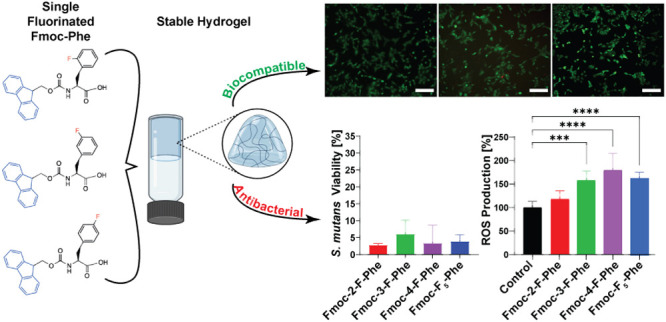

## Introduction

Antimicrobial resistance is a major global health challenge, threatening the efficacy of existing treatments and complicating the management of infections.^1,2^ The rapid rise of multidrug-resistant pathogens underscores the urgent need for innovative therapeutic strategies.^3,4^

Self-assembled nanostructures and hydrogels offer a versatile platform for combating infections while reducing antibiotic reliance.^5–8^ Hydrogels formed by self-assembly of short peptides or amino acids are cost-effective, easy to fabricate, and free of toxic cross-linking agents.^5–15^ Their antimicrobial activity arises from their nanofibrillar networks that physically disrupt bacterial membranes or create an inhospitable microenvironment for microbial growth.^6,9^ These hydrogels hold promise as they also exhibit drug delivery and wound healing properties.^5,6,16,17^

A key example of an antibacterial hydrogel is the Fmoc-F_5_-Phe, which spontaneously self-assembles into nanofibrillar structures in aqueous solution, leading to the formation of a hydrogel.^9,16,18–22^ We previously demonstrated the antibacterial activity of these assemblies, and its compatibility with mammalian cells, incorporating it into a composite resin for dental restorations, which conferred antibacterial properties against *S. mutans*.^19^ Additionally, we have shown that Fmoc-F_5_-Phe coatings significantly reduce bacterial viability and biofilm formation on surfaces, with the Fmoc-containing building block outperforming a similar analogue. Additionally, the coatings increased surface hydrophobicity, which was linked to enhanced antibacterial activity.^23^ This effect aligns with the well-known superhydrophobic nature of fluorinated molecules, as observed for Teflon and other fluorinated polymers.^24–28^ Despite its promising antibacterial properties, Fmoc-F_5_-Phe exhibits low mechanical rigidity and instability as a hydrogel, going through phase separation and collapsing after a few days of gelation. One way to address these limitations is to incorporate additional building blocks through coassembly to produce a stable hydrogel capable of retaining its properties over extended periods.^20,29,30^ However, while coassembly enhances the stability of Fmoc-F_5_-Phe hydrogel, it may also compromise its antibacterial properties.

Halogenation of the Phe aromatic ring was shown to significantly influence the self-assembly process and the characteristics of the formed hydrogel.^11,28,31–33^ Halogenation, including fluorination, alters the electronic properties of the aromatic ring by attracting electron density toward the halogen atom due to its high electronegativity. Additionally, the position of the halogen on the aromatic ring introduces steric effects, which further influence the self-assembly process by affecting molecular packing and interactions.^34^ Even minor structural variations, such as altering the position of a single atom on the aromatic ring, can greatly impact the hydrogels’ stability and overall properties.^31,34,35^

To improve the unstable yet highly antibacterial Fmoc-F_5_-Phe hydrogel, we examined single-fluorinated Fmoc-Phe derivatives. Our findings show that single-fluorinated Fmoc-Phe forms stable hydrogels, with Fmoc-4-F-Phe exhibiting antibacterial activity comparable to that of Fmoc-F_5_-Phe, even at low concentrations. These self-assembling molecular systems offer advantages over conventional antibiotics and may be used for sustained drug release, surface coating, and medical integration, positioning them as promising candidates for infection prevention. These findings provide insights into the role of fluorination in hydrogel stability and antibacterial performance, guiding the development of more effective antimicrobial materials.

## Experimental Section

### Materials

All of the fluorinated amino acids were purchased from Chem-Implex Inc. (IL) and were kept at 4 °C. Dimethyl sulfoxide (DMSO) was purchased from Sigma-Aldrich (Rehovot, Israel).

### Preparation of Fluorinated Fmoc-Phe Hydrogels

Hydrogels were prepared using the solvent switch method, as previously described.^33^ Briefly, stock solutions of the various derivatives were prepared in DMSO at a concentration of 100 mg/mL. The hydrogels were formed by diluting the stock solution with ddH_2_O, resulting in final concentrations of 0.1, 1, 2.5, and 5 mg/mL.

### Gelation Kinetics Analysis

Immediately after hydrogel preparation, 100 μL samples were placed into a 96-well plate. Absorbance of the samples was measured at 350 nm using a TECAN Infinite M200PRO plate reader for 10 h.

### Scanning Electron Microscopy (SEM)

A small sample from each hydrogel was placed on an aluminum stub using a spatula. The samples were frozen at −80 °C for 2 h. All liquids were diminished by freeze-drying. The samples were then sputtered by an 8 nm gold layer and analyzed using a JSM-IT100, JEOL scanning electron microscope (Tokyo, Japan).

### Transmission Electron Microscopy (TEM)

10 μL of hydrogel was placed on a 400-mesh copper grid (Electron Microscopy Sciences, Ltd.), and the excess liquid was discarded after 1 min. The samples were negatively stained using 10 μL of 2% uranyl acetate for 1 min and imaged with a JEOL 1200EX transmission electron microscope.

### Rheological Analysis

*In situ* hydrogel formation, storage and loss moduli, and kinetics were characterized by using an AR-G2 rheometer (TA Instruments). Time-sweep oscillatory tests were performed using a 20 mm parallel plate geometry on 220 μL fresh solution (resulting in a gap size of 0.6 mm) at room temperature, in triplicate. Oscillatory strain (0.01–100%) and frequency sweeps (0.1–10 Hz) were conducted to find the linear viscoelastic region, in which the time-sweep oscillatory tests were performed. *G*′ and *G*″, the storage and loss moduli, respectively, were obtained at 5 Hz oscillation and 0.5% strain deformation for each sample. The gelation kinetics, the gel’s stiffness (*G*′), and the gel’s stability were evaluated over 6 h.

### Mammalian Cell Viability Analysis

NIH 3T3 mouse fibroblast cells were cultured in Dulbecco’s modified Eagle’s medium (DMEM) supplemented with 10% fetal bovine serum (FBS) and 100 UmL^–1^ penicillin, 100 UmL^–1^ streptomycin. The cells were incubated at 37 °C in a humidified atmosphere containing 5% CO_2_. The various hydrogels were prepared as described above. 100 μL of each hydrogel was placed in each well of a 96-well plate (*n* = 5). Cells were seeded on the hydrogels at a density of 1 × 10^4^ cells/well and were incubated at 37 °C in a humidified atmosphere containing 5% CO_2_. Cell viability was assessed using the Alamar Blue assay at 1 and 3 days after seeding. At each time point, the medium was removed from the wells and a 100 μL solution of DMEM containing 10% Alamar Blue (BioRad, Israel) was added to each well, followed by a 4-h incubation. Absorbance was then measured using a Tecan Spark plate reader at 570 (reduced) and 600 nm (oxidized). The percentage of reduced Alamar Blue was calculated according to the manufacturer’s instructions. After the measurement, the Alamar Blue solution was removed from each well and replaced with a fresh medium. The cells were incubated under the same conditions until the next time point.

### Live/Dead Staining Assay

A qualitative assessment of cell viability on the hydrogels was performed using the Live/Dead staining assay. NIH 3T3 mouse fibroblast cells were seeded on the hydrogels at a density of 2 × 10^4^ cells/well and incubated for 24 h. The medium was replaced by 50 μL of Live/Dead staining solution containing fluorescein diacetate (green) (6.6 μg/mL) and propidium iodide (red) (5 μg/mL), which was used to label the live and dead cells, respectively. The cells were immediately imaged using a Leica SP8 X Confocal Microscope.

### *Streptococcus mutans* Growth Inhibition Kinetics

*S. mutans* bacteria (ATCC 35668) were kept at −80 °C. At the beginning of each experiment, a small amount was thawed and grown under anaerobic conditions in brain heart infusion (BHI) broth (BD Difco) for 24 h. The bacterial solution was then diluted to an OD_600_ of 0.01. Simultaneously, the hydrogels were prepared as outlined above. 100 μL of each hydrogel was placed in a 96-well plate, with a total of 8 repeats for each hydrogel. After full gelation of all hydrogels, 100 μL of bacterial solution was added to each hydrogel sample, and the plate was sealed to ensure anaerobic conditions. Absorbance was measured at 600 nm in the presence or absence of the hydrogels using a TECAN Infinite M200PRO plate reader for 24 h.

### Assessment of Bacterial Viability Using Live/Dead Fluorescence Staining

Following growth inhibition kinetic measurement, samples were washed with saline and incubated for 5 min in a solution containing Syto9 and propidium iodide (PI) (L13152 Live/Dead BacLight Bacterial Viability Kit, Molecular Probes, OR). Fluorescence emission was detected using an ECLIPSE E600 fluorescence microscope (Nikon, Japan).

### Evaluation of Antibacterial Activity of Hydrogels via ATP-Dependent Luminescence Assay

Microbial cell viability was also quantified based on bacterial ATP production, used to convert beetle luciferin to oxyluciferin and light using the BacTiter Glo Microbial viability assay kit (Promega, G8231). *S. mutans* bacteria were exposed to the various hydrogels, as described in the previous paragraph. After a 24 h incubation under anaerobic conditions, a 50 μL sample was transferred from each well to a new well, and an equal volume of the BacTiter solution was added. Luminescence was immediately measured using a Glomax Navigator-Luminometer.

### Antibacterial Activity of Fluorinated Fmoc-Phe Derivatives in Their Monomeric Form

*S. mutans* cultures were diluted to an OD_600_ of 0.01. Simultaneously, stock solutions of the fluorinated Fmoc-Phe derivatives were diluted in 0.07% Tween 80 to prevent self-assembly and to maintain the amino acids in their monomeric form. Aliquots of 100 μL of each diluted derivative were added to a 96-well plate at final concentrations of 0.1, 0.01, and 0.001 mg/mL (*n* = 6). Then, 100 μL of the bacterial suspension was added to each well, and the plate was sealed to maintain anaerobic conditions. Bacterial growth kinetics were monitored by measuring absorbance at 600 nm over 10 h using a TECAN Infinite M200PRO plate reader. Untreated bacteria and bacteria exposed to 0.07% Tween 80 in ddH_2_O served as negative and vehicle controls, respectively.

### Assessment of Anti-Biofilm Activity of Fluorinated Fmoc-Phe Derivatives

*S. mutans* was cultured on the fluorinated Fmoc-Phe derivatives for 48 h in BHI medium supplemented with 1% sucrose, under anaerobic conditions. To promote biofilm formation, the medium was replaced with fresh sucrose-supplemented BHI after 24 h. Following 48 h of incubation, the medium was removed, and the samples were gently washed with PBS to remove nonadherent (planktonic) bacteria. Wells were then stained with fluorescein isothiocyanate (FITC)-conjugated lectin from *Triticum vulgaris* (WGA) by incubating at room temperature for 30 min. After staining, samples were washed twice with PBS, and the fluorescence intensity was measured at 485 nm excitation and 520 nm emission using a TECAN Infinite M200PRO plate reader.

### High-Resolution Scanning Electron Microscopy (HRSEM) Imaging of the Bacteria on the Hydrogels

Following 24 h exposure to the hydrogels, bacterial samples were washed three times in PBS and fixed using 2.5% glutaraldehyde in PBS for 1 h at room temperature. The samples were then washed three times in PBS, followed by a dehydration series with ethanol at 25%, 50%, 75%, and 90%. The samples were then left in 100% ethanol for 30 min and placed onto glass coverslips, followed by critical point drying. The samples were then sputtered with an 8 nm gold layer. Micrographs were recorded using a Zeiss GeminiSEM 300 – HRSEM (Anon, Carl Zeiss, Germany).

### Quantification of Reactive Oxygen Species (ROS) in *S. mutans* Following Treatment with Hydrogels

*S. mutans* were grown overnight and diluted to an OD_600_ of 0.1. The bacteria were incubated with 2′,7′–dichlorofluorescin diacetate (DCFDA) for 30 min prior to seeding. For the assay, 100 μL of each hydrogel was cast into a 96-well plate with 9 replicates per hydrogel formulation. Following complete gelation, 100 μL of the DCFDA-labeled bacterial suspension was added to each well. The plate was sealed to maintain the anaerobic conditions. Untreated bacteria in a BHI medium served as the negative control. Fluorescence measurements were performed using a TECAN Infinite M200PRO plate reader, which was preheated to 37 °C. ROS production was monitored kinetically over 4 h, with excitation at 485 nm and emission at 535 nm.

### Statistical Analysis

Data was processed using the GraphPad Prism 10.4.1 software. Statistical significance was examined using *t* test and one-way ANOVA to determine the *p*-value. *p* < 0.05 was considered a statistically significant difference.

## Results and Discussion

### Kinetic Analysis, Morphology, and Mechanical Characterization of Fluorinated Fmoc-Phe Hydrogels

Single-fluorinated Fmoc-Phe derivatives possess the ability to spontaneously assemble into ordered nanostructures that result in the formation of hydrogels. Here, we investigated four derivatives: Three derivatives, each containing a single fluorine atom in a varying position, namely, Fmoc-2-F-Phe, Fmoc-3-F-Phe, and Fmoc-4-F-Phe, and Fmoc-F_5_-Phe, which contains five fluorine atoms (Figure 1a–d). First, we examined the tendency of each derivative to form structures using the solvent switch method with DMSO as the organic solvent. SEM and TEM analyses revealed a fibrillary microstructure produced by all derivatives (Figure 1e–h for SEM and Figure 1i–l for TEM). Although all four derivatives formed fibrous nanostructures, differences could be observed in their microstructures. Fmoc-3-F-Phe and Fmoc-4-F-Phe gave rise to robust fibrils characterized by an average diameter of 122 and 98 nm, respectively. In contrast, Fmoc-2-F-Phe and Fmoc-F_5_-Phe displayed significantly thinner fibrils, measuring approximately 25 and 20 nm in diameter, respectively (Figures 1i–l and S1).

**Figure 1:**
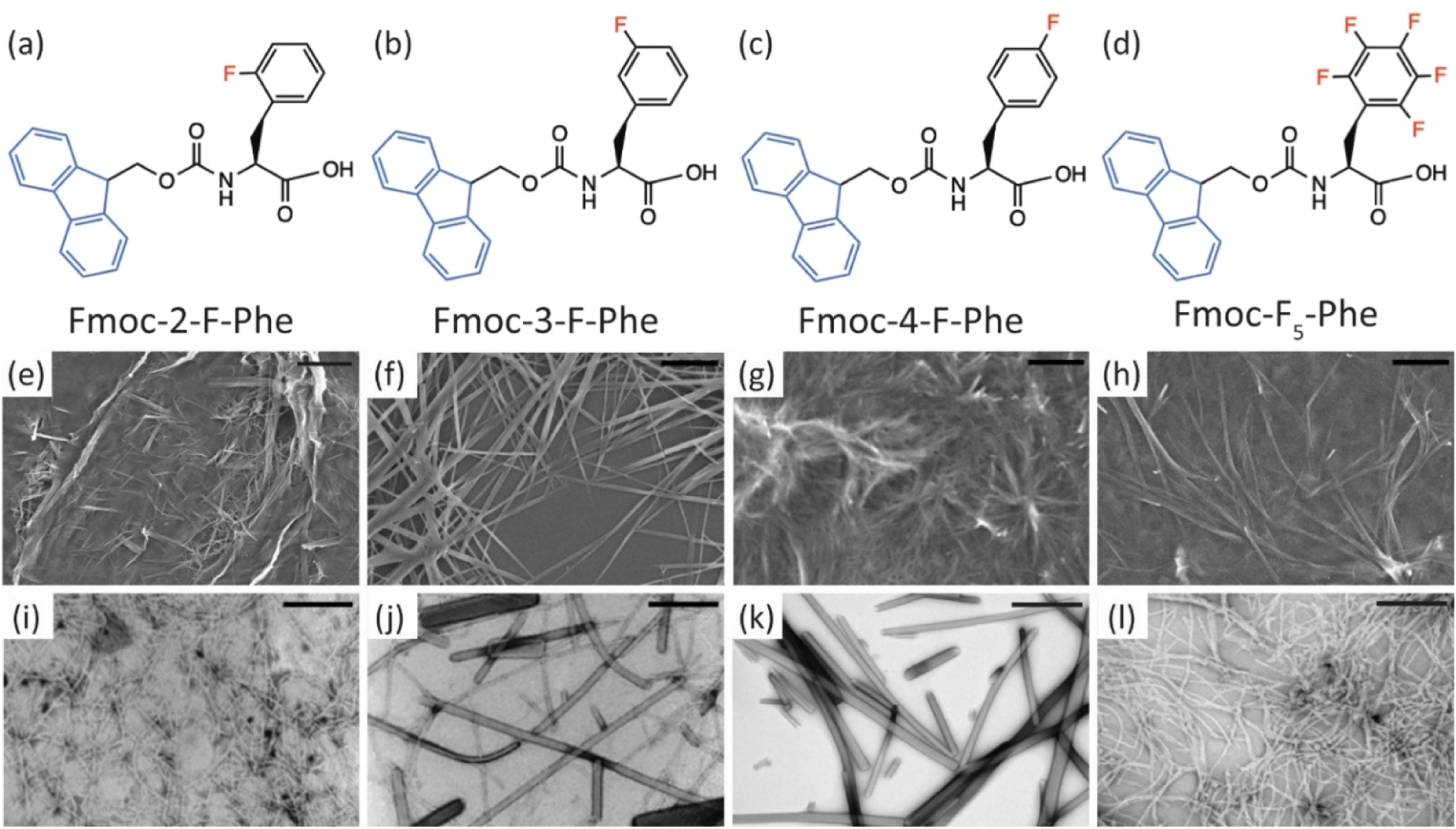
Chemical structures and electron microscopy characterization of fluorinated Fmoc-Phe derivatives. (a–d) Chemical structures of the building blocks. (e–h) SEM images of the formed structures, scale bar is 10 μm. (i–l) TEM images of the formed structures, scale bar is 1 μm. (a, e, i) Fmoc-2-F-Phe, (b, f, j) Fmoc-3-F-Phe, (c, g, k) Fmoc-4-F-Phe, and (d, h, l) Fmoc-F_5_-Phe.

When using the solvent switch method, the gelation mechanism typically involves a transition in the solution’s optical properties, where a turbid solution transforms into a clearer hydrogel as organized nanostructures emerge from low-molecular-weight building blocks. While Fmoc-4-F-Phe formed an opaque hydrogel, Fmoc-2-F-Phe, Fmoc-3-F-Phe, and Fmoc-F_5_-Phe formed transparent hydrogels after 1 h (Figure 2a, left). However, the Fmoc-F_5_-Phe hydrogel was not stable, and collapsed after a few days, whereas all of the other fluorinated derivatives were stable over time and maintained a clear 3D self-supporting hydrogel structure (Figure 2a, right). These findings suggest that fluorination influences both fibril morphology and hydrogel transparency, with thinner fibrils generally correlating with increased transparency. However, excessive fluorination, as in Fmoc-F_5_-Phe, appears to compromise the hydrogel stability.

**Figure 2:**
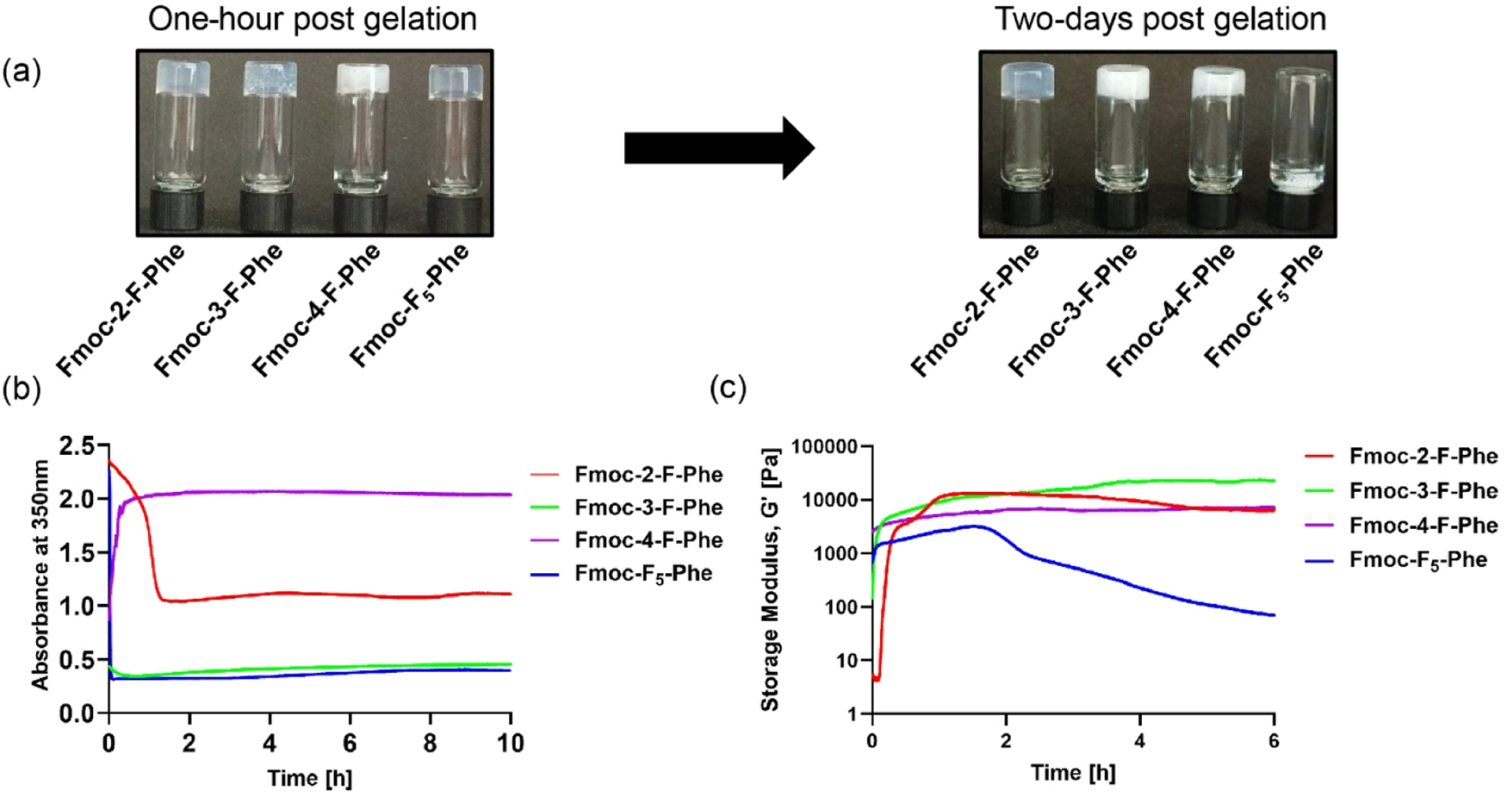
Kinetic and rheological characterization of the assemblies. (a) Hydrogels formed by fluorinated Fmoc-Phe derivatives 1 h post gelation (left) and 2 days post gelation (right). Vials from left to right: Fmoc-2-F-Phe, Fmoc-3-F-Phe, Fmoc-4-F-Phe, and Fmoc-F_5_-Phe. (b) OD kinetics at 350 nm for the first 10 h of hydrogel formation (*n* = 3). (c) *In situ* time-sweep oscillation measurements of the storage modulus (*n* = 3).

To examine the kinetics of the self-assembly process leading to nanostructure formation, we continuously assessed the solution turbidity at a wavelength of 350 nm over 10 h (Figure 2b). Notably, the Fmoc-F_5_-Phe and Fmoc-3-F-Phe hydrogels exhibited the most rapid decline in the OD values, indicative of their rapid gelation process. Conversely, the Fmoc-2-F-Phe hydrogel initially displayed a very high opacity with an OD of almost 2.5 a.u, but over time, it progressively became more transparent, reaching saturation after ∼90 min (Figure 2b). In contrast, the absorbance of the Fmoc-4-F-Phe hydrogel gradually increased over time, eventually stabilizing on an OD of ∼2 a.u, signifying an opposite transition from a transparent to an opaque hydrogel.

Rheological analysis was further performed to evaluate the mechanical properties of the hydrogels. First, dynamic strain sweep (0.01–100% strain) and frequency sweep (0.1–10 Hz) oscillatory measurements were performed to identify the appropriate conditions for gelation analysis (Figure S2). Based on the frequency sweep and oscillatory strain sweep analysis, the *in situ* kinetics of the hydrogels formation and their rheological properties were characterized by time sweep measurements at a fixed strain of 0.5% and frequency of 5 Hz, over 6 h (Figure 2c, and Table 1). The storage modulus, *G*′, was significantly higher than the loss modulus, *G*″, for all hydrogels, which is indicative of a viscoelastic gel. In all cases, the viscoelastic properties of the hydrogels, Tan δ (*G*″/*G*′) values after gelation, were <0.1, indicating stable hydrogel formation (Figure S3).

**Table 1:** Rheological Characterization of Hydrogels Formed by Fluorinated Fmoc-Phe Derivatives

	Fmoc-2-F-Phe	Fmoc-3-F-Phe	Fmoc-4-F-Phe	Fmoc-F_5_-Phe
highest *G*′ value [kPa]	13.5	23.3	7.3	3.2
time to reach highest *G*′ [min]	94	346	360	91
*G*′ at 100 min/highest *G*′ [%]	100	51	81	94
*G*′ at 360 min/highest *G*′ [%]	48	97	100	2

All hydrogels showed an increase in the storage modulus over time, corresponding to the self-assembly process and nanostructure formation. However, each hydrogel exhibited different gelation kinetics and different rheological parameters. Fmoc-3-F-Phe exhibited the highest storage modulus of 23.3 kPa ∼ 6 h after hydrogel formation (Figure 2c and Table 1). Although the highest value was obtained after 6 h, a short time of only 7 min was required for its *G*′ values to overtake all other hydrogels. The Fmoc-4-F-Phe hydrogel showed a similar behavior, where the highest *G*′ value was obtained after 6 h, but with a lower value of 7.3 kPa. In contrast, the Fmoc-F_5_-Phe hydrogel exhibited a different behavior of an initial increase in the storage modulus with a peak after 90 min, followed by a sudden decrease, indicating its low stability. Fmoc-F_5_-Phe reached its highest *G*′ value of 3.2 kPa after 91 min and dropped sharply to 2% after 6 h (Table 1). Fmoc-2-F-Phe and Fmoc-F_5_-Phe, which display limited hydrogel stability, also demonstrate similar low fiber dimensions detected using microscopy imaging (Figures 2 and S1).

### Biocompatibility Evaluation of the Fluorinated Fmoc-Phe Hydrogels

To further study the potential of the fluorinated Fmoc-Phe hydrogels to be used as scaffolds for tissue engineering applications, we tested their biocompatibility using *in vitro* cell culture experiments. NIH 3T3 fibroblast cells were seeded on various hydrogels. The viability of the fibroblasts was evaluated using Live/Dead staining (Figure 3a–e) and a quantitative Alamar Blue viability assay after 1 and 3 days (Figure 3f).

**Figure 3:**
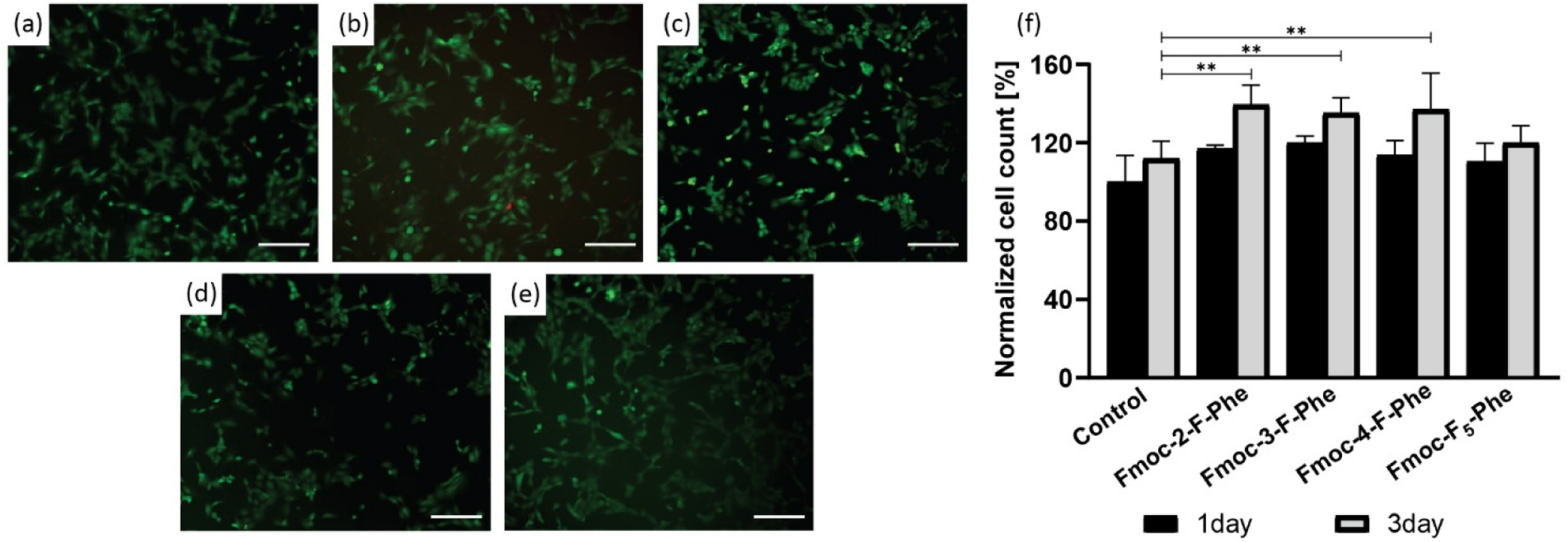
NIH 3T3 fibroblast cell viability in the presence of the hydrogels. (a–e) Live/Dead fluorescent staining assay after overnight growth on: (a) Control, (b) Fmoc-2-F-Phe, (c) Fmoc-3-F-Phe, (d) Fmoc-4-F-Phe, and (e) Fmoc-F_5_-Phe. Images are the merged staining of both fluorescein diacetate (green, indicating live cells) and propidium iodide (red, indicating dead cells), Scale bar is 25 μm. (f) Quantitative cell viability using Alamar Blue assay following 1 day and 3 days of growth on the hydrogels (*n* = 5). **p < 0.01, measured using one-way ANOVA.

All formulations appear to be biocompatible when compared to the control, with several even demonstrating enhanced NIH 3T3 proliferation (*p* < 0.01). This improvement may be due to the ability of certain formulations to provide a more supportive 3D scaffold, potentially offering favorable surface interactions and structural cues that promote fibroblast adhesion and proliferation (Figure 3).

### Antibacterial Characterization of the Fluorinated Fmoc-Phe Hydrogels

Altering the position of fluorine on the aromatic ring was shown to impact the physicochemical characteristics of the hydrogel.^34–36^ We further examined the antibacterial properties of the hydrogels. Fmoc-F_5_-Phe was previously reported for its antibacterial activity against several types of bacteria.^18,19^ Here, we evaluated whether single-fluorinated Fmoc-Phe molecules present a similar effect. For this purpose, *S. mutans* bacteria were exposed to several concentrations of various fluorinated Fmoc-Phe hydrogels overnight. The hydrogels’ antibacterial activity was evaluated by kinetic growth analysis (Figure 4a–c) and by quantitative measurement of ATP production (Figure 4d–f). Bacteria cultured in BHI broth with DMSO at the same concentration as in the hydrogel served as a control in both assays.

**Figure 4:**
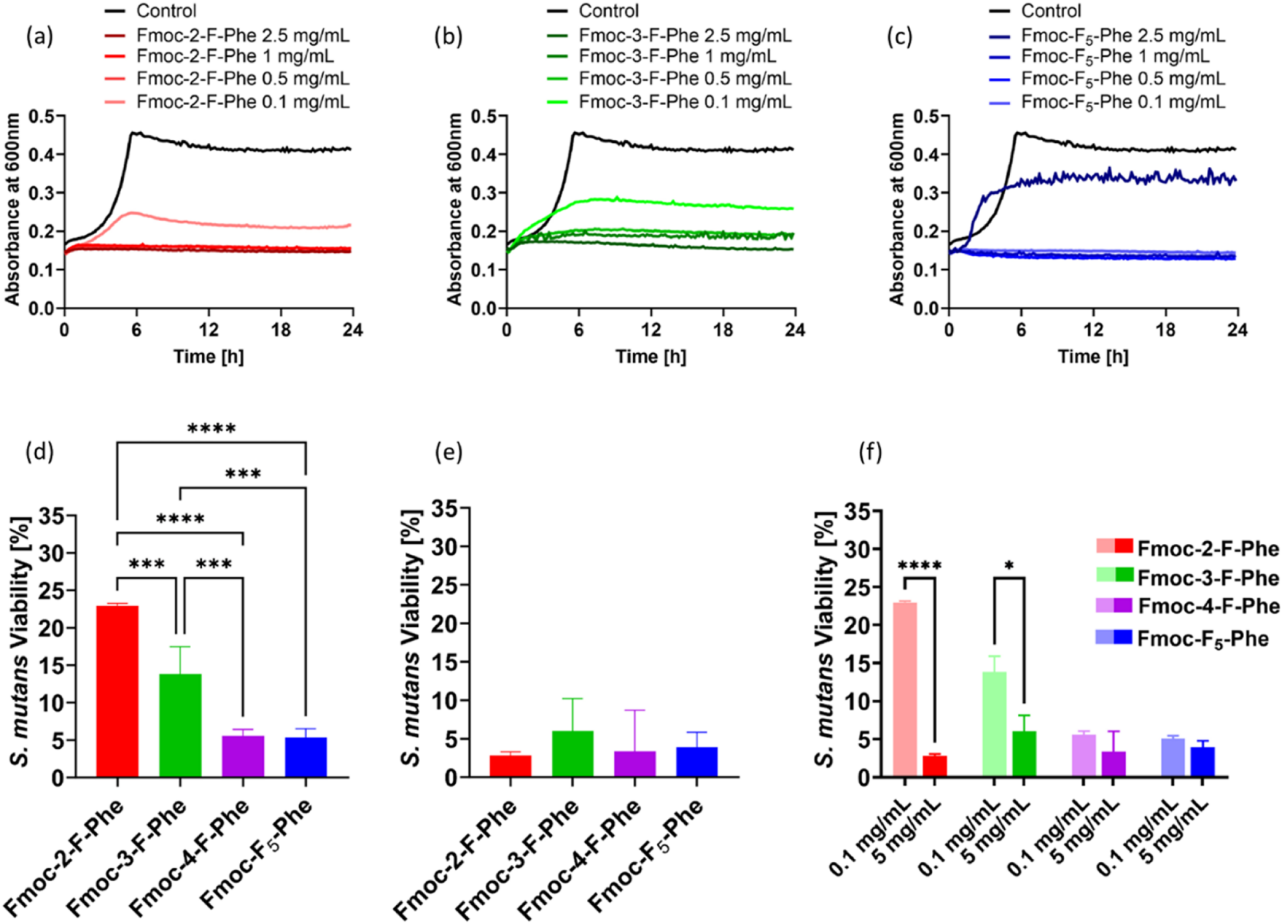
Antibacterial activity of fluorinated Fmoc-Phe hydrogels against *S. mutans*. (a–c) Bacterial growth kinetics evaluated by turbidity analysis via absorbance readings at 600 nm. *S. mutans* bacteria were exposed to the hydrogels at various concentrations, as indicated: (a) Fmoc-2-F-Phe, (b) Fmoc-3-F-Phe, and (c) Fmoc-F_5_-Phe. (d–f) Quantification of bacterial viability following 24 h exposure to the various hydrogels at different concentrations measured by luminescence (*n* = 5): (d) 0.1 mg/mL, (e) 5 mg/mL, (f) 5 mg/mL compared to 0.1 mg/mL. *p < 0.05, **p < 0.01, ***p < 0.001, ****p < 0.0001 as measured using (d, e) one-way ANOVA, and (f) T-Test.

The fluorinated Fmoc-Phe hydrogels exhibited significant antibacterial activity against *S. mutans*, starting in the early log phase, and completely inhibited bacterial growth. The antibacterial activity of the Fmoc-2-F-Phe and Fmoc-3-F-Phe hydrogels was found to be dose-dependent, exhibiting partial bacterial inhibition at lower concentrations and increased antibacterial activity with the increase in concentration (Figure 4a,b). In contrast, Fmoc-F_5_-Phe exhibited complete inhibition, even at the lowest tested concentration (0.1 mg/mL). However, at 2.5 mg/mL, it displayed high opacity, which did not correlate with the effects observed at lower concentrations. This discrepancy can be attributed to the turbid nature of the hydrogel at higher concentrations. Since a turbid hydrogel absorbs light regardless of bacterial growth, the standard absorbance-based assay may not be reliable for evaluating bacterial inhibition in Fmoc-F_5_-Phe at high concentrations (Figure 4c). Similarly, Fmoc-4-F-Phe formed a turbid hydrogel even at lower concentrations, making light absorption analysis unsuitable for assessing bacterial growth under these conditions (Figure S4). To overcome the difficulty in measuring the antibacterial activity of Fmoc-4-F-Phe, a luminescence test was performed, assessing the amount of ATP produced by viable bacteria in the samples (Figure 4d–f). Exposure to low concentrations of the hydrogels (0.1 mg/mL) revealed substantial differences between the hydrogels, as indicated by ATP production. Fmoc-2-F-Phe showed the least antibacterial activity (77% viability reduction), followed by Fmoc-3-F-Phe (86% reduction), and finally Fmoc-4-F-Phe, which showed a substantial reduction of approximately 95% in bacterial viability, similar to Fmoc-F_5_-Phe (Figure 4d). At 5 mg/mL, the hydrogels exhibited antibacterial activity across all derivatives with no significant differences between the groups (Figure 4e). A comparison between the two concentrations of the fluorinated Fmoc-Phe hydrogels is presented in Figure 4f, confirming that Fmoc-2-F-Phe and Fmoc-3-F-Phe exerted a dose-dependent effect on bacterial viability, while Fmoc-4-F-Phe and Fmoc-F_5_-Phe similarly showed much more substantial antibacterial activity.

Surprisingly, the position of only one fluorine atom in the Fmoc-Phe molecule significantly affects the properties of the hydrogel, including its antibacterial activity. Furthermore, it is shown that a single fluorine substituted at position four can produce an antibacterial effect similar to that of five fluorine atoms, even though their physicochemical properties are distinctly different.

As an additional qualitative measure, bacterial viability was evaluated using the Live/Dead assay following 24 h exposure to the fluorinated Fmoc-Phe hydrogels. This assay utilizes SYTO9, a green-fluorescent nucleic acid stain that penetrates both live and dead bacteria, and propidium iodide (PI), a red fluorescent dye that enters only bacteria with compromised membranes, typically indicating dead or membrane-damaged cells. Staining of bacteria with SYTO9 and PI generally supported the trends observed in the quantitative assays, demonstrating antibacterial properties, including the dose-dependent effects of Fmoc-2-F-Phe and Fmoc-3-F-Phe on the bacteria (Figure S5).

Notably, Fmoc-4-F-Phe treatment showed a strong nonspecific red dye, apparently due to absorption of the dye to the 4-F-Phe fibrils (Figure S5), limiting the interpretability of its results in this assay. However, no green dye staining was detected for the Fmoc-4-F-Phe samples at all concentrations, indicating no live bacteria, thus suggesting its strong antibacterial effect.

To understand whether the self-assembly of the fluorinated molecules into ordered structures is responsible for the antibacterial properties, we incubated the fluorinated building blocks in the presence of Tween 80 to inhibit fibril formation. TEM analysis revealed no visible assemblies under these conditions, even at the highest tested concentration of the fluorinated building blocks of 0.1 mg/mL. Higher fluorinated building blocks concentration required a higher Tween 80 concentration, which is toxic to the bacteria. At concentrations of 0.001 and 0.01 mg/mL of the fluorinated building block, no antibacterial properties were shown, except for Fmoc-2-F-Phe, which demonstrated mild antibacterial activity. However, despite the absence of detectable nanostructures at a concentration of 0.1 mg/mL, the fluorinated building blocks with Tween samples exhibited antibacterial activity (Figure S6). This observation may indicate the presence of protofibrils or ultrasmall aggregates below the resolution limit of TEM, or alternatively suggest an additional direct antibacterial mechanism exerted by the monomeric species. However, the monomeric form likely lacks the structural integrity required for scaffold-based applications. Thus, the self-assembled hydrogel state remains the most suitable and functional form for antibacterial biomaterial development.

To evaluate the ability of *S. mutans* to adhere to the fluorinated hydrogels and to form biofilms, we cultured the bacteria on the different Fmoc-Phe derivatives and on empty well plates, as a control, for 48 h. Biofilm formation was then assessed using FITC-conjugated wheat germ agglutinin (WGA), a lectin that specifically binds to N-acetylglucosamine. This polysaccharide is an abundant component of *S. mutans* biofilm and traditionally used to assess bacterial biofilm formation^37–39^ (Figure 5a). Fluorescence intensity measurements revealed a marked reduction in biofilm formation on the fluorinated hydrogels compared with the control, suggesting that the nanostructured assemblies not only reduced bacterial viability but also impaired biofilm development, a key factor in pathogenicity (Figure 5b).

**Figure 5:**
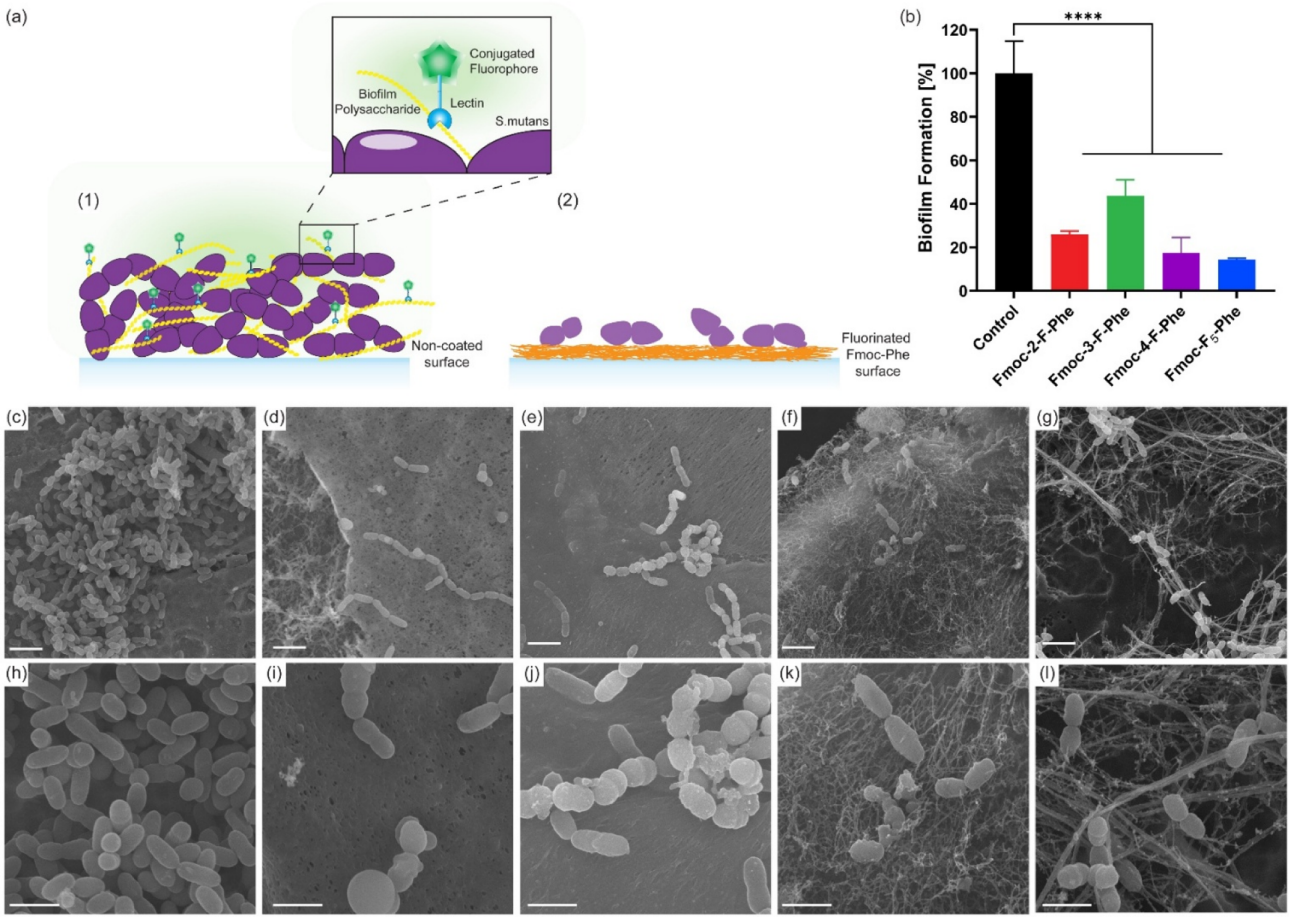
Inhibition of *S. mutans* biofilm formation on fluorinated hydrogels. (a) Schematic representation of biofilm formation and inhibition (a1) biofilm formation and exopolysaccharide detection on an uncoated surface using FITC-conjugated lectin, and (a2) impaired bacterial integrity and reduced biofilm formation on fluorinated Fmoc-Phe hydrogels. (b) Quantification of exopolysaccharides in the biofilm using fluorescence intensity of lectin bound to polysaccharides. (c–l) HRSEM images of *S. mutans* cultured on the various hydrogels; (c, h) Control, (d, i) Fmoc-2-F-Phe, (e, j) Fmoc-3-F-Phe, (f, k) Fmoc-4-F-Phe, (g, l) Fmoc-F_5_-Phe. Scale Bars are (c–g) 2 μm and (h–l) 1 μm.

To further investigate these observations, we examined the bacterial morphology using HRSEM. Control bacteria without nanostructures exhibited a typical rod-shaped morphology, arranged in short chains, with intact and aggregated cells (Figure 5c,h). In contrast, *S. mutans* seeded on the various nanostructured hydrogels showed a marked reduction in the bacterial presence and significant morphological alterations of the bacteria (Figure 5d–g,i–l). Bacteria grown on Fmoc-2-F-Phe or Fmoc-3-F-Phe retained a short-chain arrangement but adopted a more spherical morphology (Figure 5d,e,i,j). Bacteria grown on Fmoc-4-F-Phe or Fmoc-F_5_-Phe showed notably diminished bacterial growth, with minimal evidence of chain formation (Figure 5f,g,k,l). *S. mutans* exposed to all nanostructures exhibited substantial morphological abnormalities, including irregular shapes, disrupted cell membranes, and heterogeneous sizes. These observations indicate that the various fluorinated Fmoc-Phe hydrogel derivatives not only inhibit biofilm formation but also severely compromise bacterial structural integrity.

HRSEM images also revealed a clear distinction between bacterial cells and the surrounding nanofibrillar networks. These mesh-like structures are consistent with the self-assembled morphology of the fluorinated amino-acid derivatives rather than with cellular debris (Figure S7). Notably, bacteria in contact with the hydrogels appeared structurally compromised, often isolated, deformed, or shrunken, compared to the healthy, intact cells observed in the untreated group. These notable morphological differences support the notion that the hydrogels physically disrupt the bacterial surface, contributing to reduced viability, as observed in the quantitative assays. Since the hydrogels were applied as surface coatings and did not diffuse into the bulk medium, bacterial exposure was limited to direct contact with the hydrogel surface. Thus, the morphological damage observed in *S. mutans* attached to the hydrogel surfaces reflects the gels’ capacity to interfere with initial bacterial adhesion and early biofilm formation. This model is especially relevant for oral applications, where initial surface colonization is a critical first step in biofilm development.^7,40^ Morphological changes in *S. mutans* related to the growth medium have been previously reported, including transitions between spherical and rod-shaped cells and reductions in the cell length under acidic conditions. Such changes are likely attributed to stress-induced modifications in cell wall synthesis and maintenance.^41–43^

To further assess the bacterial stress response following exposure to the fluorinated hydrogels, the production of reactive oxygen species (ROS) was measured. ROS production is a well-established marker of bacterial stress and an early indicator of membrane disruption and metabolic dysregulation.^44,45^ Using the DCFDA assay, we found that all fluorinated Fmoc-Phe derivatives, except Fmoc-2-F-Phe, induced an increase in ROS levels compared to the untreated control (Figure 6). Notably, Fmoc-4-F-Phe and Fmoc-F_5_-Phe triggered the highest ROS production in correlation with their more pronounced morphological changes observed via HRSEM imaging. While the increase in ROS levels suggests that hydrogels not only compromise bacterial membrane integrity but also promote intracellular oxidative stress, which may contribute to the observed antibacterial activity. The differences in ROS induction among the fluorinated derivatives further support a structure–activity relationship, where the position and number of fluorine substitutions influence the materials’ ability to disrupt bacterial homeostasis. These results strengthen the proposed antibacterial mechanism involving both direct physical disruption of the bacterial membrane and chemical induction of oxidative stress.

**Figure 6:**
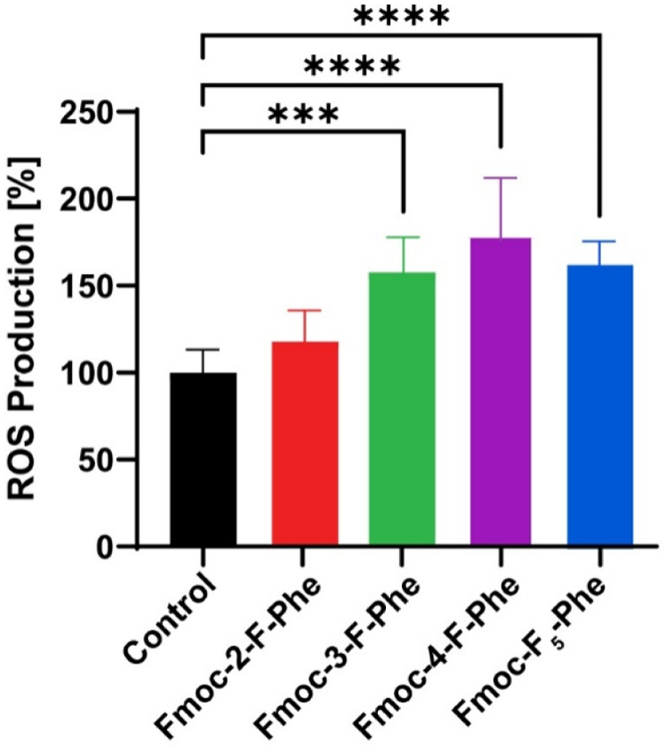
Induction of bacterial ROS by fluorinated Fmoc-Phe derivatives. Bacterial ROS levels were quantified using the DCFDA assay after incubation with Fmoc-2-F-Phe, Fmoc-3-F-Phe, Fmoc-4-F-Phe, and Fmoc-F_5_-Phe (*n* = 5). Statistical significance was determined by one-way ANOVA with post hoc test; ***p < 0.001, ****p < 0.0001.

These findings highlight the importance of understanding hydrogel-bacteria interactions for developing effective antibacterial materials. Modifying hydrogel surface properties or incorporating functional groups, such as fluorine, could further enhance bacterial disruption and improve the hydrogel performance. The antibacterial effects observed in this study highlight the potential of minimalistic peptide-mimetic building blocks. Unlike conventional antimicrobial peptides (AMPs), which are typically composed of complex multiamino acid sequences, the fluorinated Fmoc-Phe derivatives presented here demonstrate that membrane-disruptive antibacterial activity can be achieved with ultrashort, even monoamino acid–based, self-assembling systems. This minimalistic approach aligns with recent developments in AMP-inspired materials^46,47^ but offers key advantages, including easier synthesis, site-specific tunability through fluorination, and greater chemical stability. Compared to more complex self-assembling host-defense peptides reported in recent literature,^46,47^ the derivatives described in this study not only form bioactive nanostructures but also generate stable hydrogels, which may further enhance their applicability in biomedical contexts such as localized antimicrobial coatings or injectable barriers. These findings highlight the utility of rational, minimalistic molecular design in developing effective and versatile antibacterial platforms.

## Conclusions

This study examined single-fluorinated Fmoc-Phe derivatives to understand how fluorine positioning affects their self-assembly, mechanical properties, and antibacterial activity. All derivatives formed nanostructures and stable hydrogels, except for Fmoc-F_5_-Phe, which collapsed within hours. Rheological analysis revealed that Fmoc-3-F-Phe showed the highest storage modulus and that Fmoc-4-F-Phe also exhibited a relatively high and stable storage modulus. In contrast, Fmoc-F_5_-Phe showed poor stability and weak mechanical properties.

Antibacterial analysis showed that Fmoc-4-F-Phe possessed the most potent activity, maintaining efficacy at lower concentrations and inducing notable morphological disruption in bacteria. Importantly, Fmoc-4-F-Phe achieved antibacterial effects comparable to those of Fmoc-F_5_-Phe, yet with significantly improved hydrogel stability, positioning it as a more favorable candidate for biomedical use. Furthermore, all fluorinated hydrogel derivatives displayed biocompatibility with mammalian cells, underscoring their potential for future biomedical applications.

## Supplementary Material



## Notes

TEM images and fiber diameters of fluorinated Fmoc-Phe derivatives nanoassemblies (Figure S1), rheological characterization including frequency and strain sweeps (Figure S2), tan (δ) values of hydrogels (Figure S3), OD kinetics at 600 nm of Fmoc-4-F-Phe hydrogel (Figure S4), Live/Dead staining of *S. mutans* exposed to various amino-acid concentrations in their fibrillar structure (Figure S5), *S. mutans* growth kinetics following exposure to various amino-acid concentrations in their monomeric form (Figure S6), HRSEM images of the 3D structures formed by the self-assembled fluorinated amino acids (Figure S7) (PDF)
