# Supplementary material for: Positional Fluorination of Fmoc-Phenylalanine Modulates Hydrogel Structure and Antibacterial Activity
Source: Biomacromolecules. 2025 Aug 19;26(9):5705–14. doi: 10.1021/acs.biomac.5c00481 (PMC12421518; doi:10.1021/acs.biomac.5c00481)
Supplement: Supplementary file 1 [file bm5c00481_si_001.pdf]

Supporting Information

Positional Fluorination of Fmoc-Phenylalanine Modulates Hydrogel Structure and Antibacterial Activity

Ofir Doitch [1,2,3‡], Noam Rattner[1,2,3‡], Dana Cohen-Gerassi[1,2,3,4], Yoav Dan[1,2,3], Sigal Rencus-Lazar[1,2,3], Moran Aviv[1,2,3,5], and Lihi Adler-Abramovich[1,2,3*]*

[1]Department of Oral Biology, The Goldschleger School of Dental Medicine, Gray Faculty of Medical and Health Sciences, Tel Aviv University, Tel Aviv 6997801, Israel.

[2]Jan Koum Center for Nanoscience and Nanotechnology, Tel Aviv University, Tel Aviv 6997801, Israel.

[3]The Center for the Physics and Chemistry of Living Systems, Tel Aviv University, Tel Aviv 6997801, Israel.

[4]Department of Materials Science and Engineering, Tel Aviv University, Tel Aviv 6997801, Israel.

[5]School of Mechanical Engineering, Afeka Tel Aviv Academic College of Engineering, Tel Aviv 6910717, Israel.

[*]Corresponding authors: lihia@tauex.tau.ac.il; morana@afeka.ac.il

[‡]O.D. and N.R. contributed equally to this work.

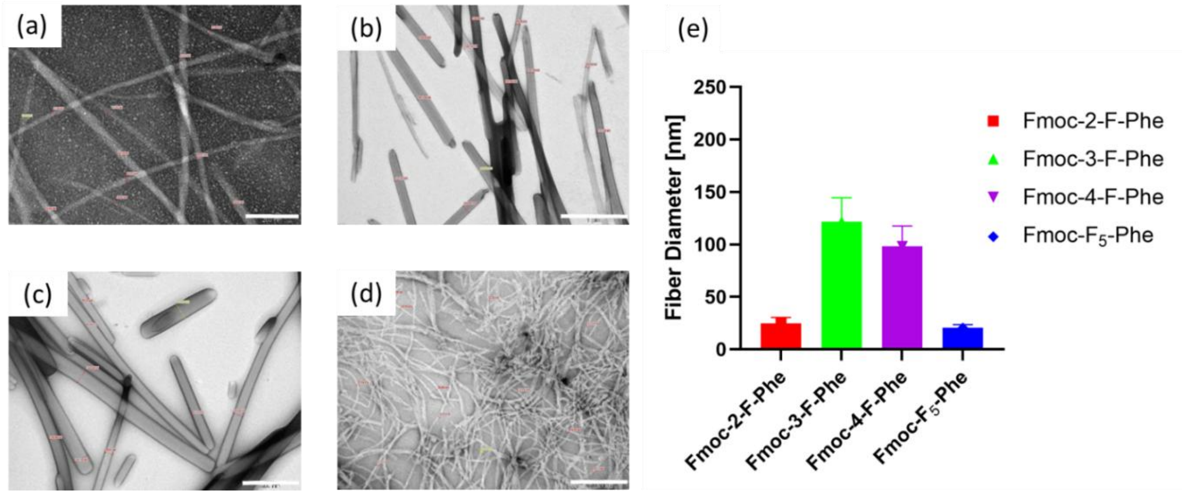

Figure S1. TEM images and fiber diameters analysis of the fluorinated Fmoc-Phe derivatives nano-assemblies. (a) Fmoc-2-F-Phe, scale bar 200 nm. (b) Fmoc-3-F-Phe, scale bar 1 µm. (c) Fmoc-4-F-Phe, scale bar 500 nm. (d) Fmoc-F_5-Phe, scale bar 500 nm. (e) Average fiber diameter.

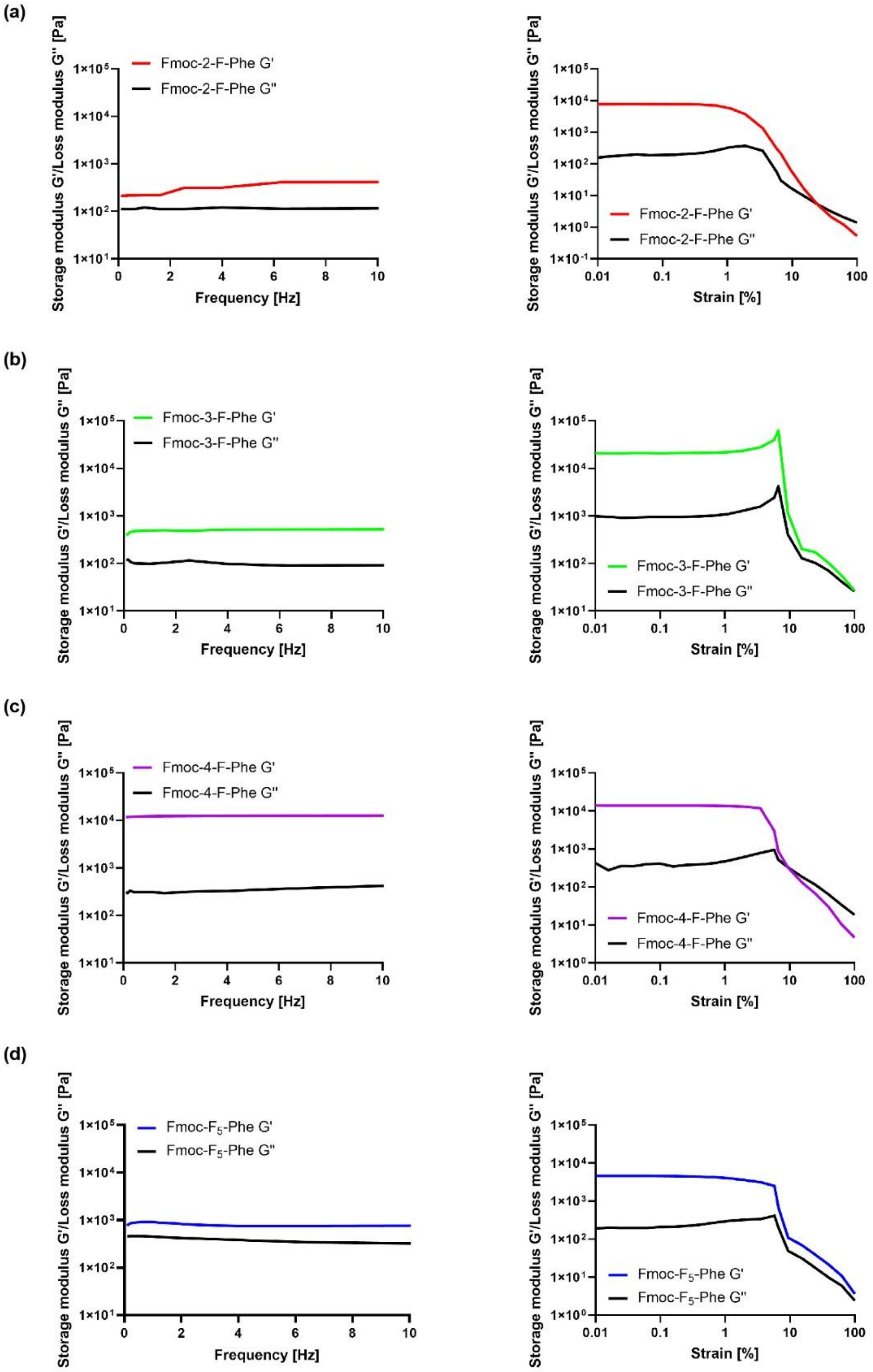

Figure S2. Rheological characterization of the hydrogels. Frequency sweep at 0.5% strain (Left) and strain sweep at 5 Hz (Right) of (a) Fmoc-2-F-Phe, (b) Fmoc-3-F-Phe, (c) Fmoc-4-F-Phe, (d) Fmoc-F_5-Phe.

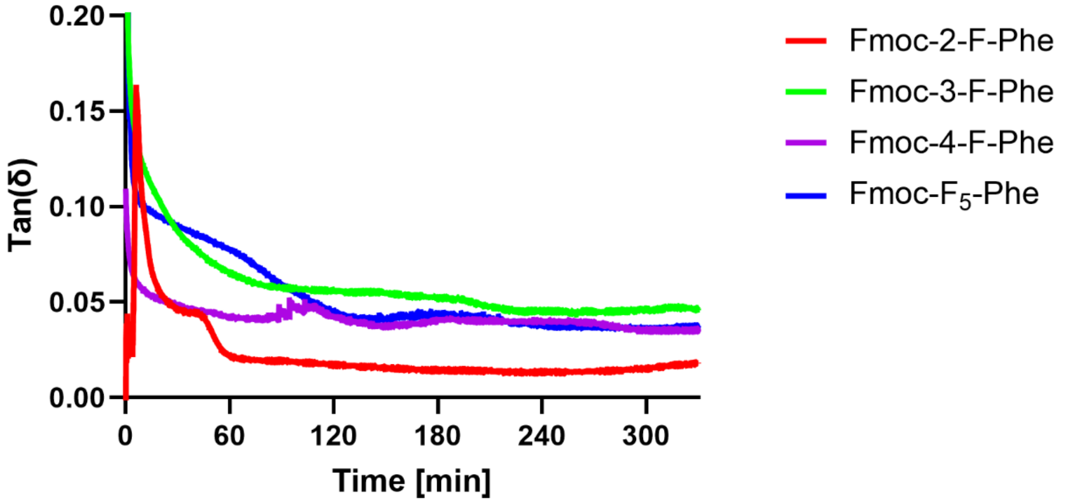

Figure S3. Viscoelastic properties of the hydrogels. Tan (δ) (G''/G') values of the various fluorinated Fmoc-Phe hydrogels.

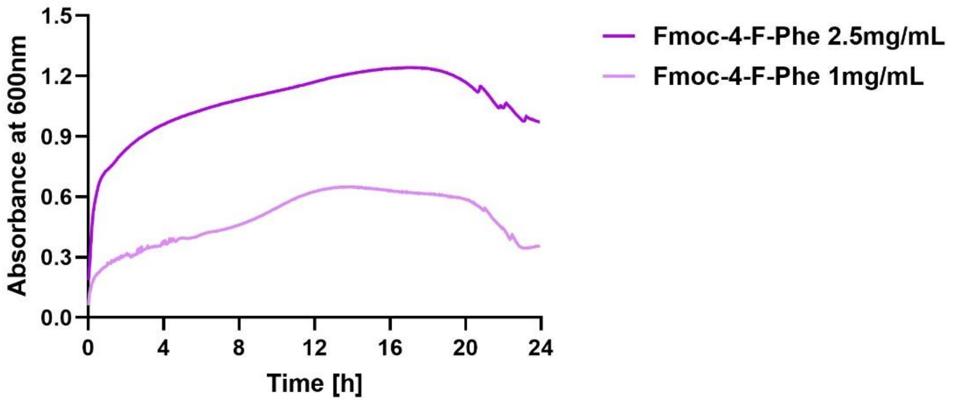

Figure S4. OD kinetics at 600 nm of the Fmoc-4-F-Phe hydrogel.

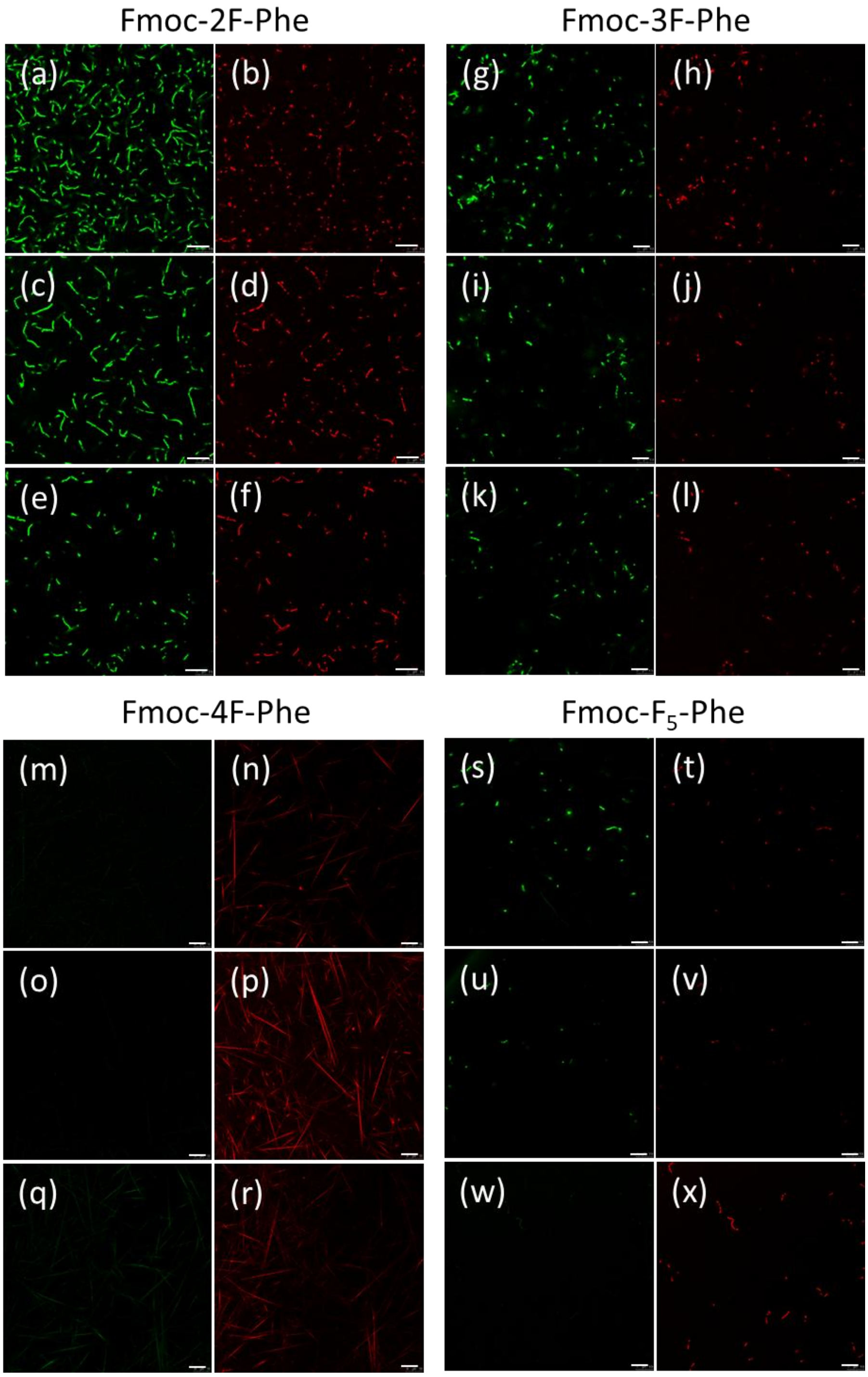

Figure S5. Bacterial viability evaluation by Live/Dead staining following overnight exposure to the fluorinated Fmoc-Phe derivatives at various concentrations. Green fluorescence of the Syto9 probe indicates bacterial cells with an intact membrane, while the red fluorescence of propidium iodide (PI) indicates dead bacterial cells. (a-f) Fmoc-2F-Phe at; (a,b) 0.5 mg/mL, (c,d) 1 mg/mL, (e,f) 2.5 mg/mL (g-l) Fmoc-3F-Phe at; (g,h) 0.5 mg/mL, (i,j) 1 mg/mL, (k,l) 2.5 mg/mL, (m-r) Fmoc-4F-Phe at; (m,n) 0.5 mg/mL, (o,p) 1 mg/mL, (q,r) 2.5 mg/mL, (s-x) Fmoc-F_5-Phe at; (s,t) 0.5 mg/mL, (u,v) 1 mg/mL, (w,x) 2.5 mg/mL. Scale bars are 10μm.

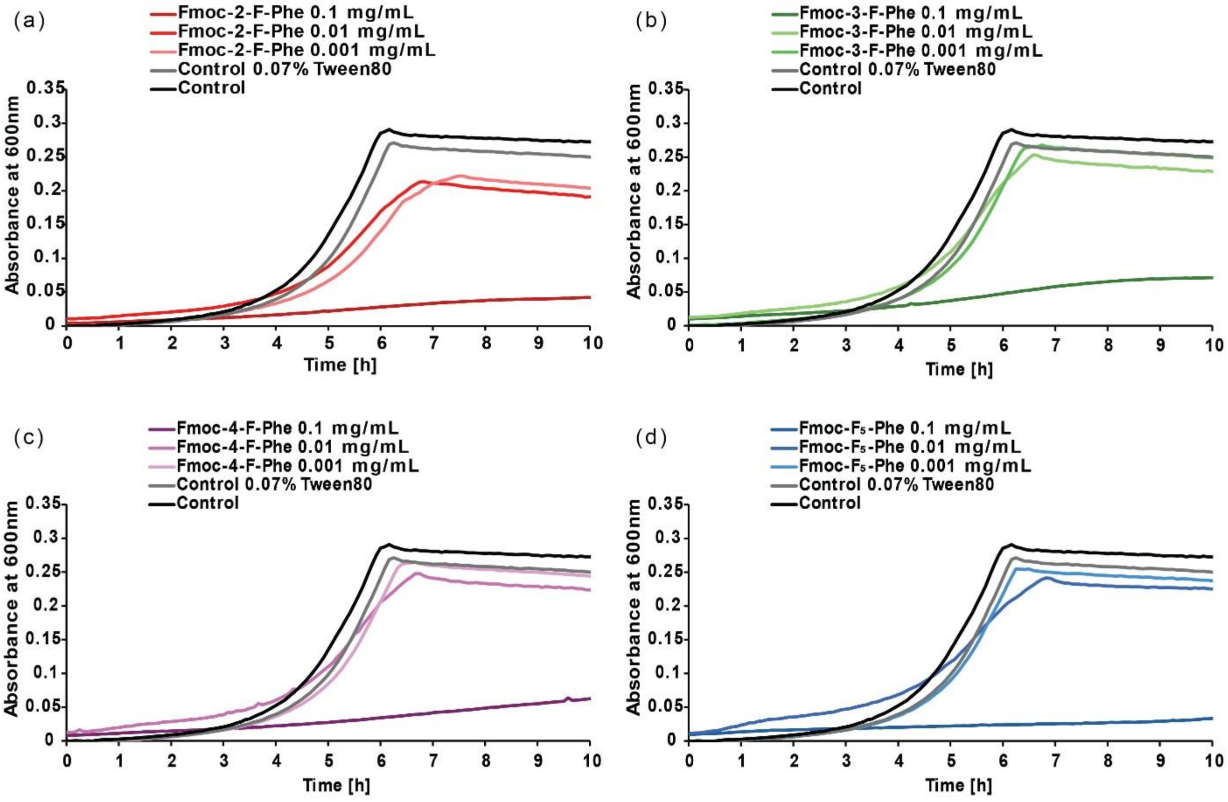

Figure S6. Bacterial growth inhibition kinetics curve during exposure to different concentrations of the fluorinated derivatives in their monomeric form. (a) Fmoc-2-F-Phe, (b) Fmoc-3-F-Phe, (c) Fmoc-4-F-Phe, (d) Fmoc-F_5-Phe.

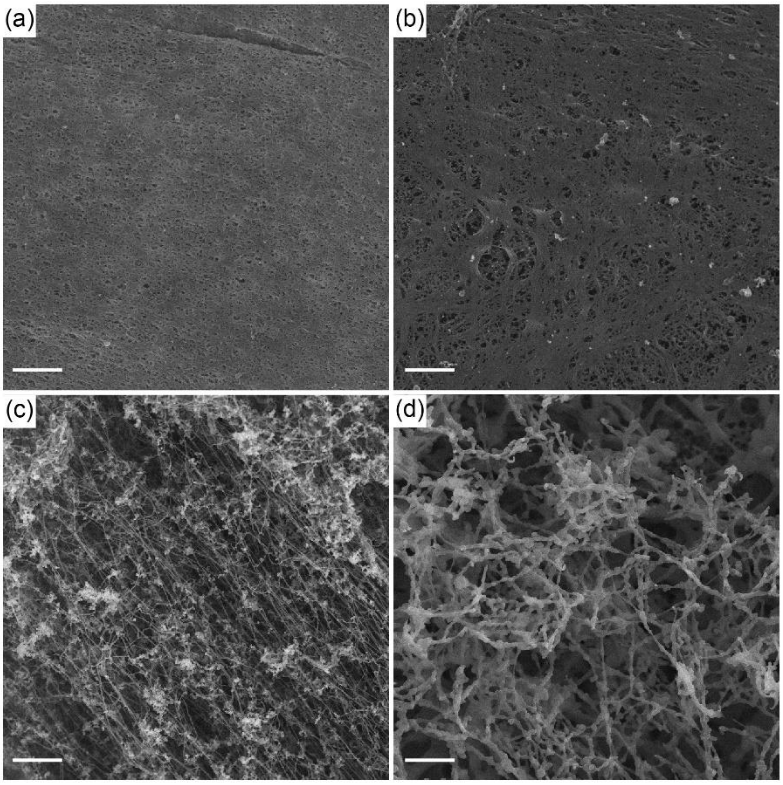

Figure S7. HRSEM images of the 3D structures formed by the self-assembled fluorinated amino acids. (a) Fmoc-2-F-Phe, (b) Fmoc-3-F-Phe, (c) Fmoc-4-F-Phe, (d) Fmoc-F_5-Phe. The scale bars are 2μm.
